# Obstructive Hydrocephalus Due to Unruptured Brain Arteriovenous Malformation: Demonstrating Transcranial Color Duplex Confirmation of Cerebral Venous Hemodynamic Alterations and Color Duplex Ultrasound Confirmation of Shunt Patency

**DOI:** 10.7759/cureus.6181

**Published:** 2019-11-18

**Authors:** Kathryn J Busch, Hosen Kiat, Alberto Avolio, Mark Butlin, Andrew Davidson

**Affiliations:** 1 Miscellaneous, Faculty of Medicine and Health Sciences, Macquarie University, Sydney, AUS; 2 Cardiology, Faculty of Medicine and Health Sciences, Macquarie University, Sydney, AUS; 3 Cardiovascular Research, Department of Biomedical Sciences, Faculty of Medicine and Health Sciences, Macquarie University, Sydney, AUS; 4 Cardiology, Department of Biomedical Sciences, Faculty of Medicine and Health Sciences, Macquarie University, Sydney, AUS; 5 Neurosurgery, Faculty of Medicine and Health Sciences, Macquarie University, Sydney, AUS

**Keywords:** transcranial color duplex, brain, arteriovenous malformation, venous, obstructive hydrocephalus, doppler

## Abstract

Given the rarity of arteriovenous malformations of the brain (bAVMs) with concomitant obstructive hydrocephalus, few papers have commented on the resultant hydrodynamic perturbations. To date, no study has specifically investigated the effect of ventricular shunting on intracranial venous parameters as measured by transcranial color duplex ultrasound (TCCD). This study investigates whether TCCD and color duplex ultrasound are useful modalities to elucidate the physiological and hemodynamic changes in a patient with bAVM following ventricular shunting.

Using TCCD, this study demonstrates that preoperatively, there is a decrease in cerebral capacitance, manifesting in a decrease in cerebral inflow and reduced venous outflow. Following shunt insertion, intracranial compliance is increased, resulting in the dilatation of previously compressed capacitance vessels and restoration of venous compliance. Color duplex ultrasound (CDU) was a useful determinant of shunt patency in the neck.

We report the first TCCD assessment of hemodynamic changes of the intracranial circulation in a patient with bAVM following ventricular-peritoneal shunting. The results lend conceptual support of a pressure gradient change with high pressure that occurs in the veins as compared to the subarachnoid space.

## Introduction

Brain AVM (bAVM) commonly presents with intracranial hemorrhage, although it may be discovered following a seizure or as an incidental finding in patients undergoing brain imaging for headache. Rarely do they cause hydrocephalus, and when they do, it is often due to obstruction of cerebrospinal fluid (CSF) pathways by a dilated venous varix [1-3].

The complex relationship between CSF flow, vascular compliance, and intracranial pressure (ICP) in the different forms of hydrocephalus is poorly understood, with the results of many studies confounded by the inclusion of both adult and pediatric patients or both communicating and obstructive hydrocephalus patients [4-5]. Very few studies of patients with hydrocephalus have investigated the relationship between vascular compliance and transcranial Doppler (TCD) parameters [6-7]; however, it has generally been reported that there is a direct relationship between the TCD pulsatility index (PI) and ICP and between the resistive index (RI) and ICP.

The effect of hydrocephalus on the intracranial venous system is poorly understood. Early animal studies suggested that hydrocephalus was associated with elevated cortical venous pressures [8], probably through a compressive phenomenon [7]. CSF shunting has been shown to result in the dilatation of cerebral veins, with an improvement in intracranial compliance, a decrease in venous resistance, and an increase in cerebral blood flow [7]. More recent MRI studies have demonstrated that NPH patients have low cortical venous pulsatility that improves with ventricular shunting [9].

Although the hemodynamic effects of bAVM on the brain have been well-described [10], the physiological effects of the obstruction of CSF pathways in the presence of bAVM have not been studied and the impact on TCD parameters is unknown. For example, although PI positively correlates with ICP, the low-resistance arteriovenous shunting that occurs in the setting of bAVM results in a reduction in PI, with larger bAVMs producing a greater increase in mean velocities and a greater decrease in PI. After the surgical removal of a bAVM, there is a gradual normalization of TCD parameters. The use of transcranial color duplex (TCCD) sonography may allow the improved visualization of the venous system in bAVM, although differentiating the draining vein from the feeding artery can be difficult.

To date, no study has specifically investigated the effect of ventricular shunting on intracranial venous parameters as measured by TCCD. This study is the first to demonstrate the physiological and hemodynamic changes in intracranial circulation in a patient with bAVM following ventricular shunting.

## Case presentation

A 54-year-old female patient presented to her general practitioner with a five-month history of persistent headache, visual blurring, and mild cognitive impairment. Her past medical and surgical history included abdominal and pelvic surgery and hip replacement surgery. She took no regular medications. She gave a history of learning difficulties throughout her life.

**Figure 1:**
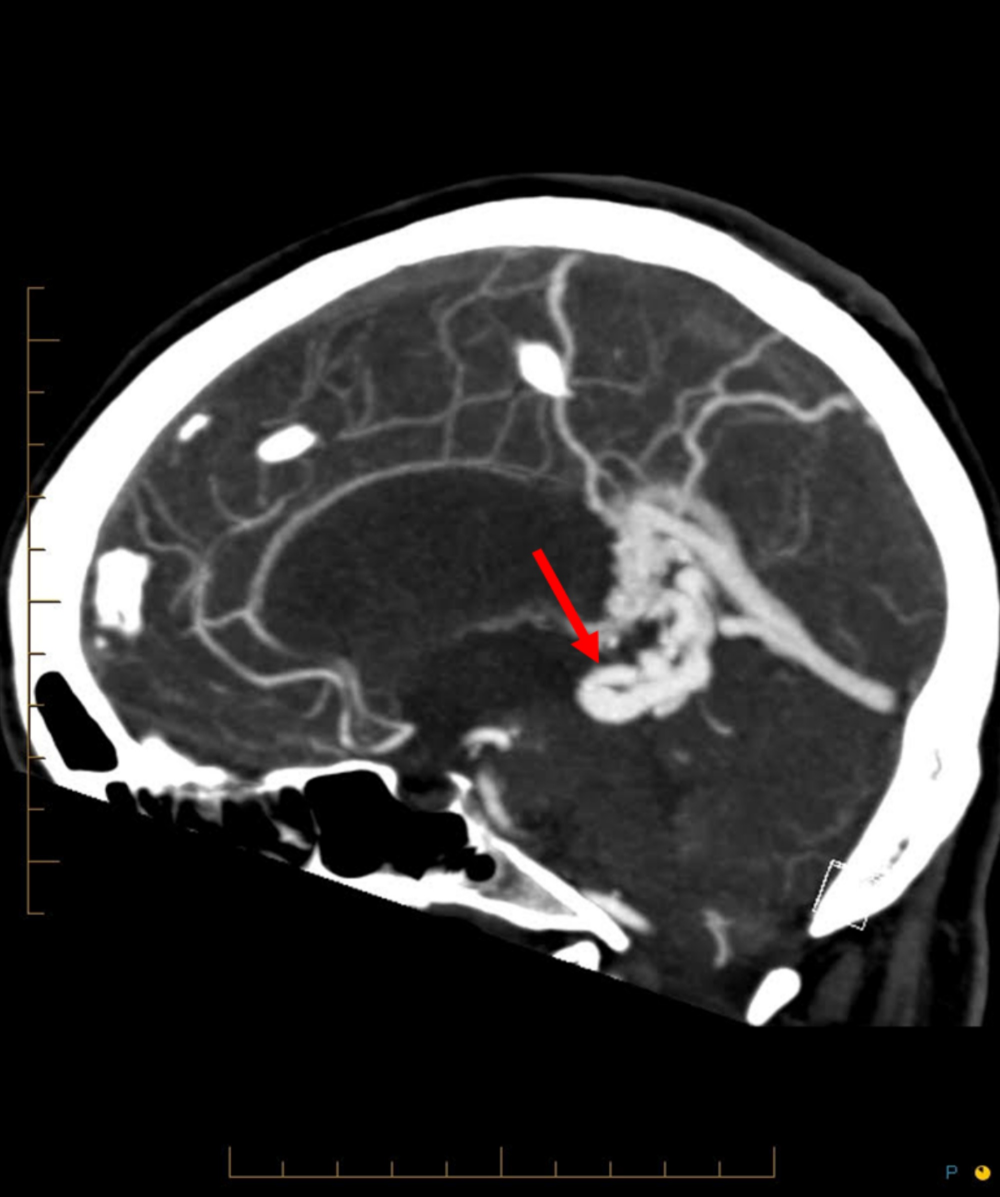
Sagittal computed tomography image of the brain The red arrow denotes where the arteriovenous malformation compresses the cerebral aqueduct.

**Figure 2:**
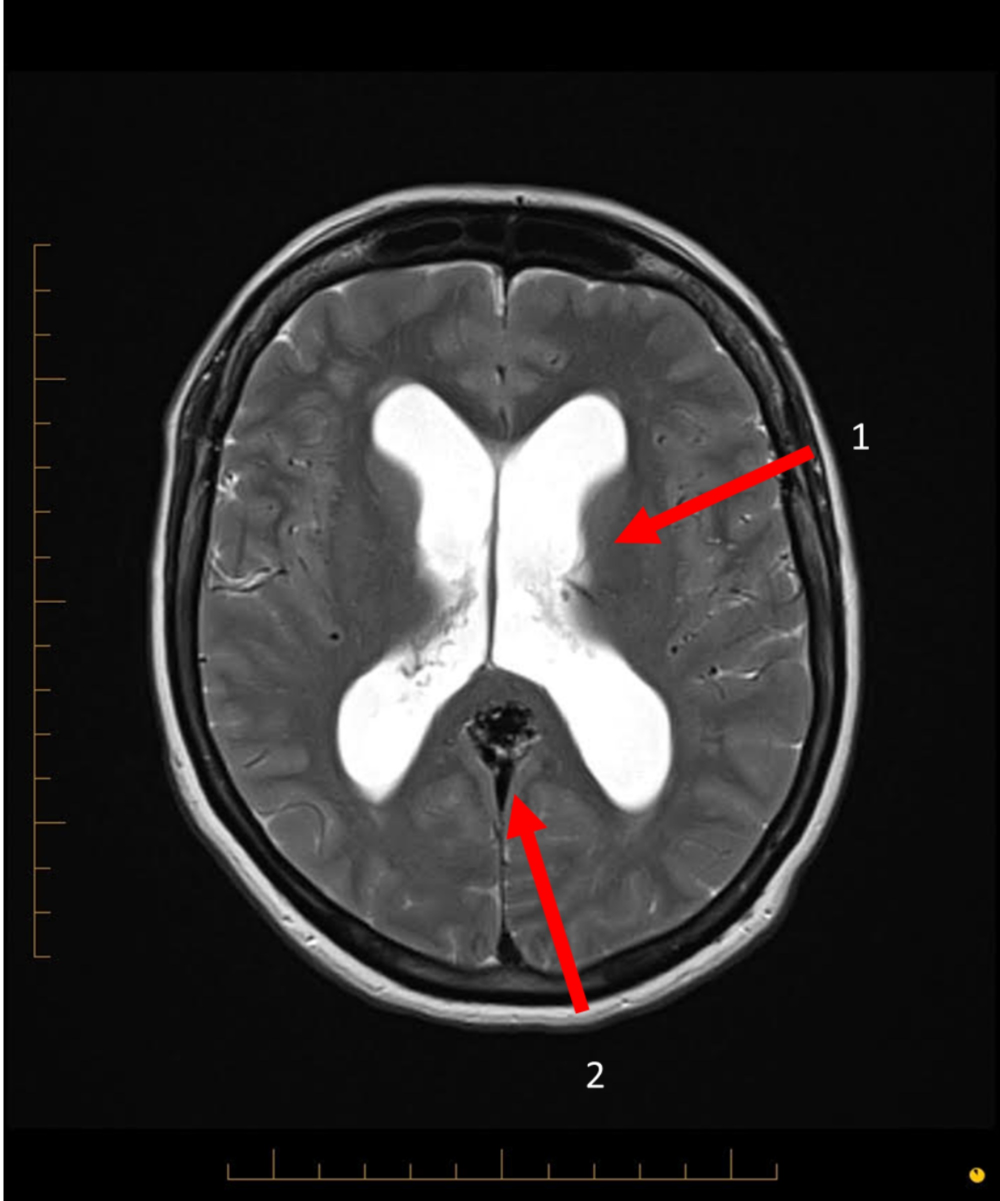
Magnetic resonance imaging axial view of the brain. Arrow 1 demonstrates the dilated hydrocephalic ventricles. Arrow 2 demonstrates the arteriovenous malformation.

Brain CT and MR imaging showed a compact 27 mm arteriovenous malformation located within the posterior interhemispheric fissure, adjacent to the splenium of the corpus callosum (Figures 1-2).

There was no evidence of previous hemorrhage. Digital subtraction angiography showed that the bAVM was associated with feeding arteries arising from the posterior cerebral arteries bilaterally, as well as from the falx and tentorial arteries. There was a network of draining veins within the pineal cistern, with a large venous varix occluding the cerebral aqueduct and causing obstructive hydrocephalus. There was deep venous drainage into the Galenic venous system. None of the bAVM was located within the eloquent brain. There was one 3 mm feeding artery aneurysm arising from the left posterior cerebral artery (PCA) and two intranidal aneurysms measuring 3 mm and 4.5 mm, respectively. Preoperative TCCD demonstrated the bilateral middle cerebral arteries (MCAs), assigned red on color duplex, denoting flow towards the probe. A brief venous signal, assigned blue, was obtained from the right Sylvian fissure representing flow direction away from the probe. No venous signal was detected from the left Sylvian fissure. The venous waveform detected on the right lacked phasicity and pulsatility. The venous signals were obtained from depths ranging from 2.7 cm to 4.2 cm.

There was no papilloedema. The formal ophthalmological assessment was normal, with no restriction of the visual field, and normal ocular coherence tomography (OCT). A neurocognitive assessment revealed mild-to-moderate impairment in cognitive tasks requiring speed and mental manipulation.

**Figure 3:**
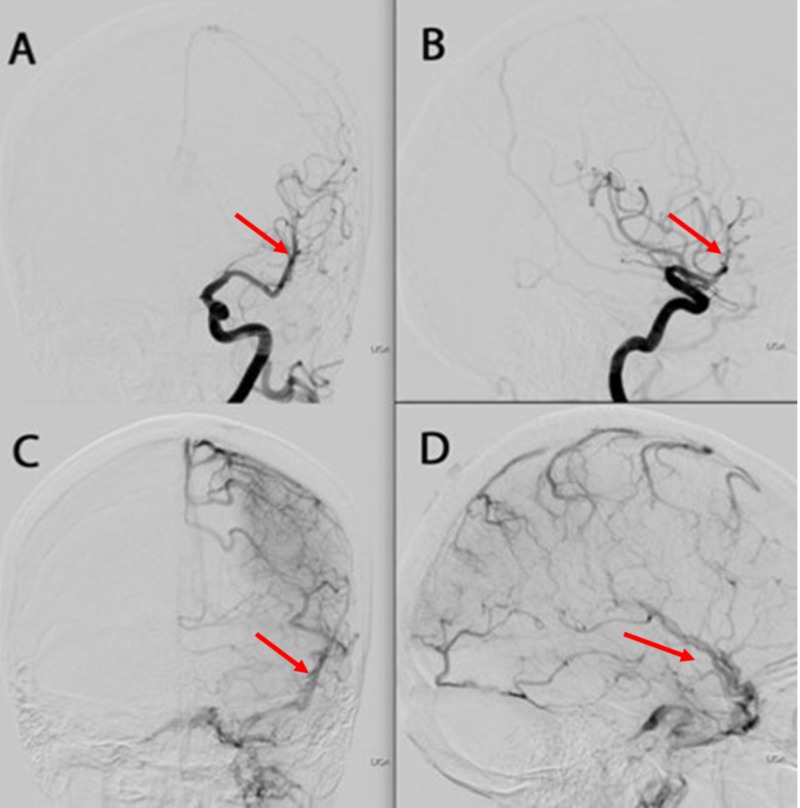
A & B = Left internal carotid artery injection, anteroposterior and lateral views, mid-arterial phase C & D = Left internal carotid artery injection, anteroposterior and lateral views, late arterial phase. The red arrows in views A & B demonstrate the left middle cerebral artery. The red arrows in views C & D demonstrate the left middle cerebral vein.

**Figure 4:**
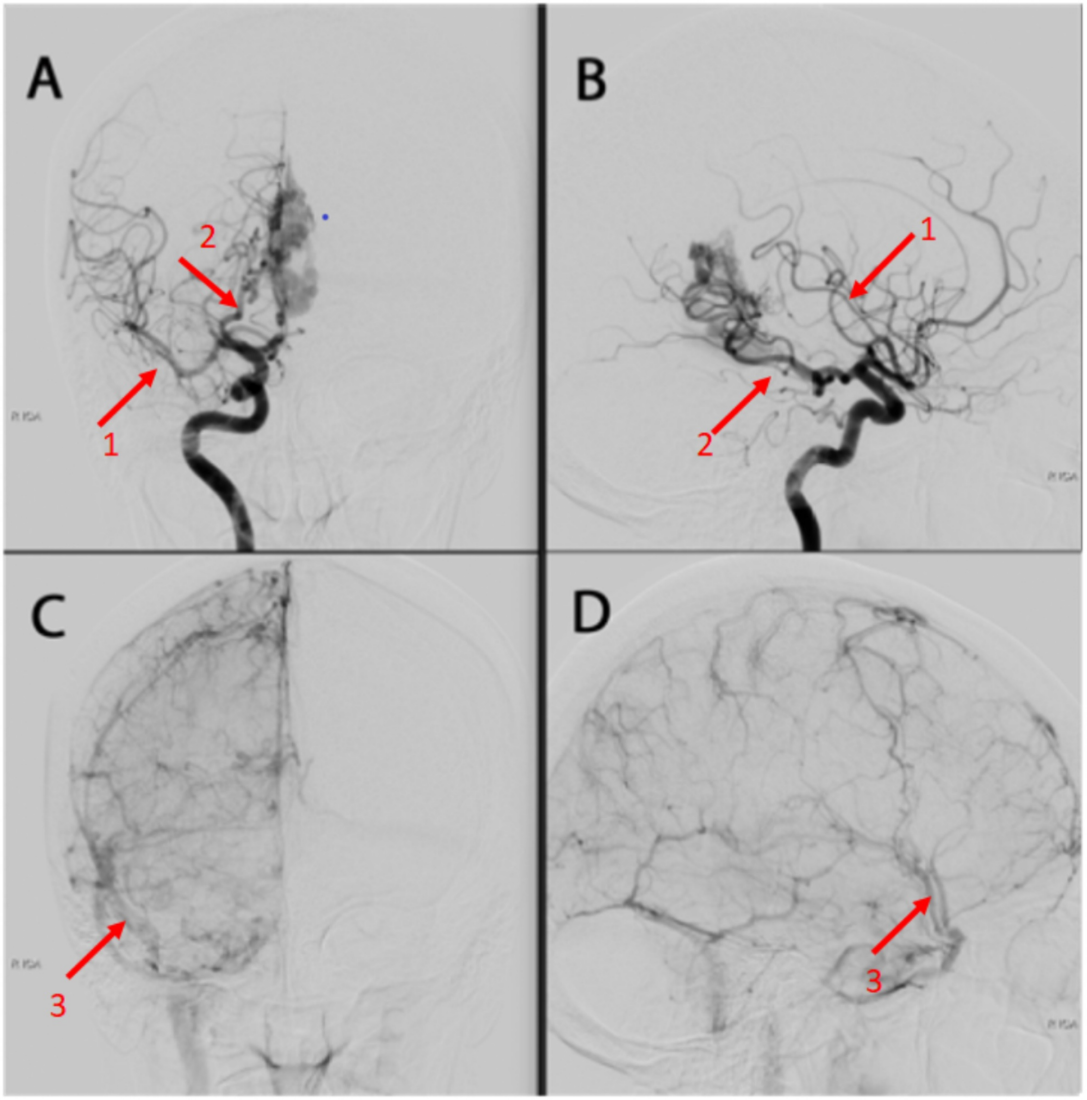
A & B = Right internal carotid artery injection, anteroposterior and lateral views, mid-arterial phase C & D = Right internal carotid artery injection, anteroposterior and lateral views, late arterial phase. Red arrows (1) in views A & B demonstrate the right middle cerebral artery. Red arrows (2) in views A & B demonstrate the posterior cerebral artery supplying the arteriovenous malformation. Red arrows (3) in views C & D represent the right middle cerebral veins.

The lesion was classified as a Spetzler-Martin grade 2, Lawton supplementary grade 6 bAVM. The patient was counseled about the risks, alternatives, and expectations of the various treatment options; it was recommended that she undergo ventriculoperitoneal shunting to treat her symptomatic hydrocephalus, followed by stereotactic radiosurgery to the bAVM nidus (Figures 3-4).

**Figure 5:**
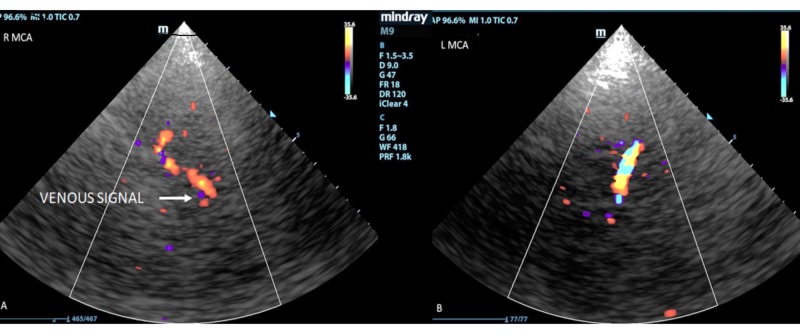
(A) Right middle cerebral artery preoperatively with no discernible venous flow. (B): Left middle cerebral artery preoperatively with no discernible venous flow.

Preoperative TCCD demonstrated the bilateral middle cerebral arteries (MCAs), assigned red on color duplex, denoting flow toward the probe. On the right, a brief venous signal was detected (assigned blue), with a non-pulsatile spectral trace. No venous signal was obtained from the left Sylvian fissure (Figures 5A-5B).

At surgery, a right frontal burrhole was used to place an antibiotic-impregnated ventricular catheter, which was inserted using neuronavigation. This was connected to a Miethke Sprung Reservoir (Christoph Miethke GmbH & Co. KG, Potsdam, Germany) and then tunneled to a right occipital incision, where it was connected to a Miethke ProGAV 2.0 programmable hydrostatic pressure valve (opening pressure set to 10 cm H_2_O) and variable gravitational-assist device (0 cm to 25 cm H2O). The distal end of the shunt tubing was inserted into the peritoneal cavity via a midline mini-laparotomy.

**Figure 6:**
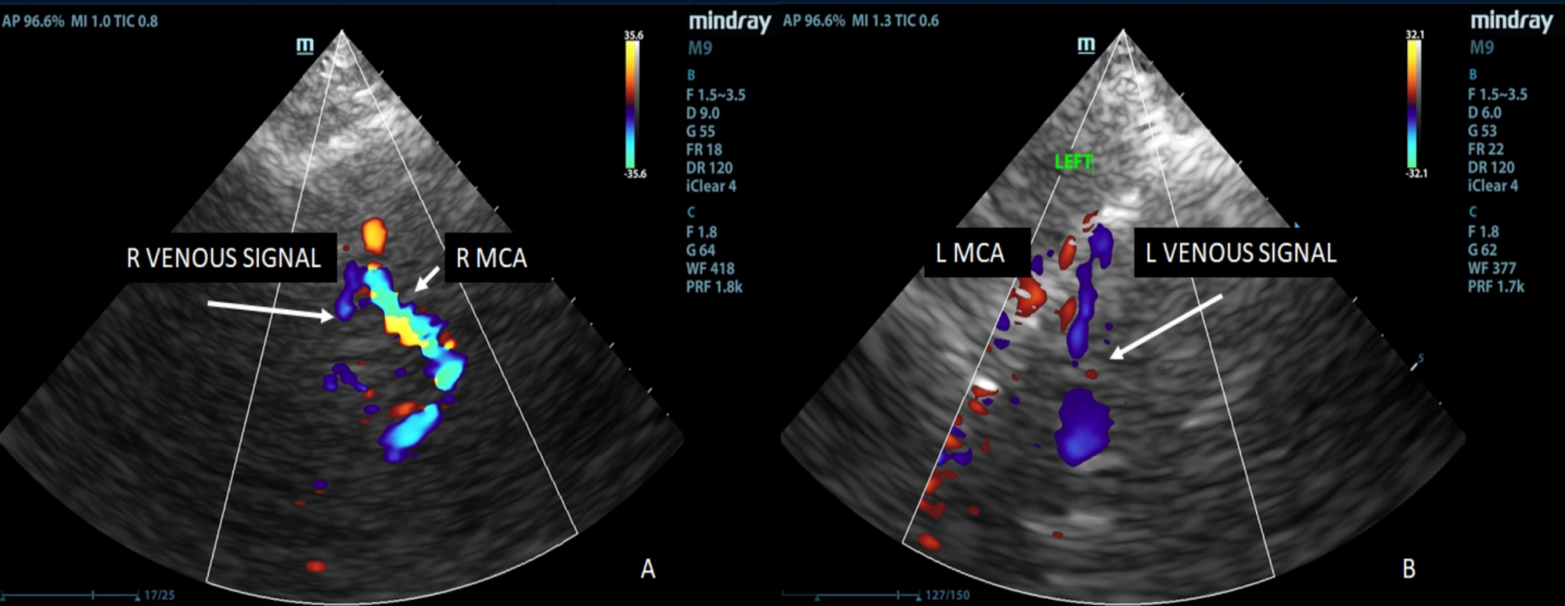
6A: Demonstrates a visible venous signal (blue) adjacent to the right middle cerebral artery (red), which could not be readily imaged pre-shunt. 6B: Demonstrates a readily discernible venous signal next to the left middle cerebral artery that was not visible prior to shunt insertion.

TCCD measurements were obtained in the intensive care unit approximately two hours following shunt insertion (Figure 6A-6B).

Two hours postoperatively, the right venous signal demonstrated a slight increase in pulsatility from 0.33 to 0.42 but visually, the color signal increased in prominence using TCCD. The left side demonstrated no visible venous signals preoperatively, however, a pulsatile spectral trace (PI =0.68) was obtained postoperatively and there was a distinct increase in the visibility and prominence of a signal using TCCD (Figure 6A, 6B).

**Figure 7:**
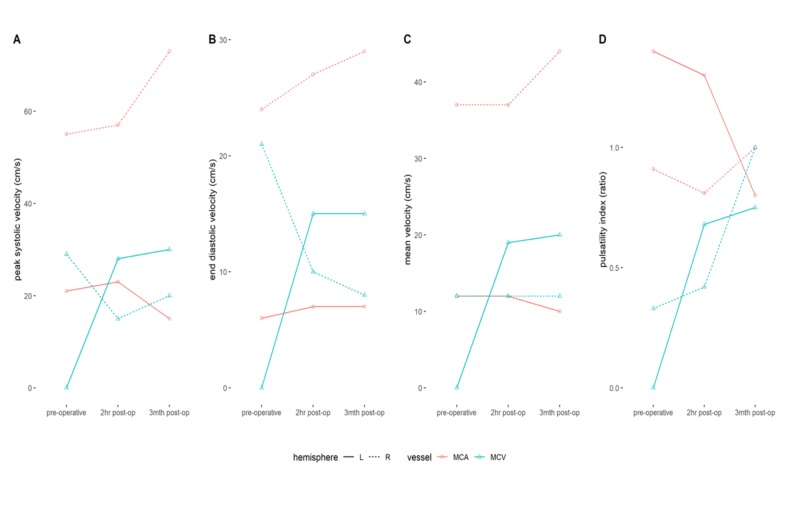
Graphical representation of the change in hemodynamic parameters from preoperative to post-shunt insertion

Middle cerebral arterial flow was lower in this patient (compared to preoperatively), with right preoperative MCA peak systolic velocity (PSV) = 55 cm/s, end-diastolic velocity (EDV) = 24 cm/s, and mean velocity = 37 cm/s. Left MCA studies showed preoperative MCA PSV = 21 cm/s, EDV = 6 cm/s, and mean velocity = 12 cm/s. There was a slight increase in postoperative velocity on the right (compared to the preoperative values) whilst the left side demonstrated little change (Figure 7).

Comparatively normal individuals have velocities twice that of this patient. Unpublished data from 20 healthy volunteers derived by the primary author have demonstrated MCA PSV of 110 ± 26 cm/s, EDV of 48 ± 11 cm/s, and mean velocity of 69 ± 15 cm/s. Normal individual MCAs have a PI of 0.92 ± 0.14.

**Figure 8:**
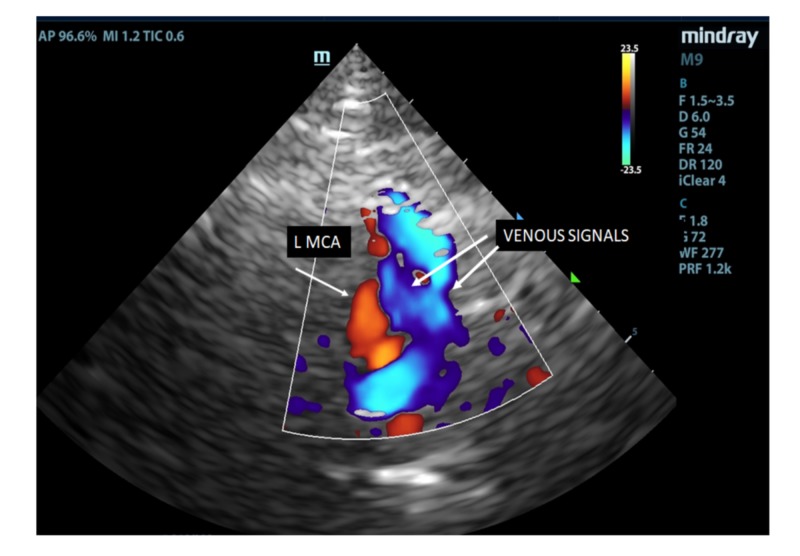
Transcranial color duplex image of the left middle cerebral artery (red) and veins within the Sylvian fissure (blue) with a further increase in prominence 3 months post shunt insertion.

At three months postoperatively, there was a further substantial increase of color flow prominence of the left middle cerebral vein (Figure 8).

**Figure 9:**
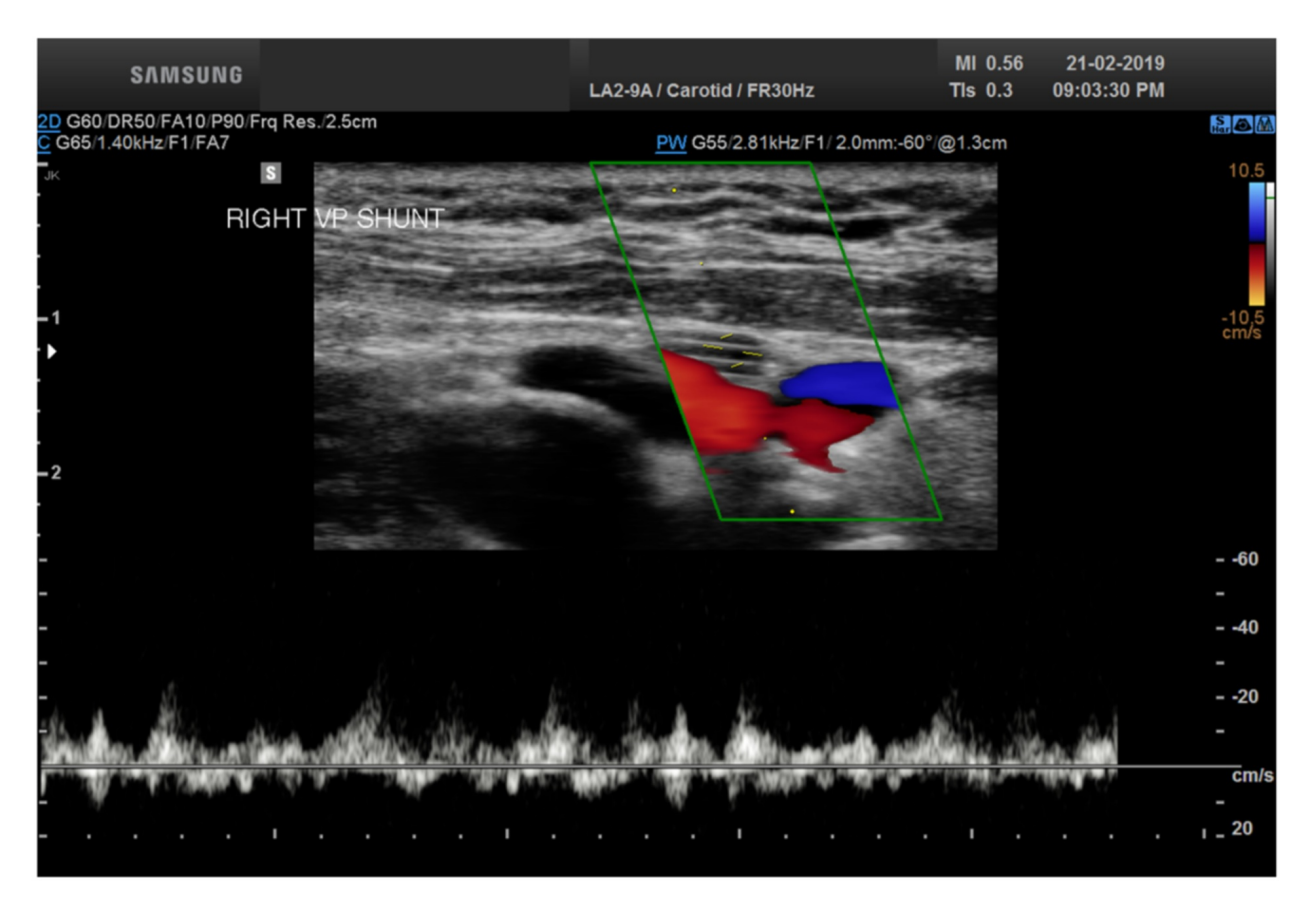
Color duplex spectral signal of cerebrospinal fluid flow within the ventriculoperitoneal shunt taken three months postoperatively. No color signal could be imaged within the shunt.

Furthermore, at the three month follow-up time interval, an attempt was made to image the VP shunt on the right side of the neck using conventional color duplex imaging and a high-resolution 2-9 MHz transducer. This attempt successfully revealed CSF flow waveforms within the shunt (Figure 5). The color flow was not readily discernible within the shunt, however, given that spectral Doppler is more sensitive than color, a spectral signal could be obtained (Figure 9).

The patient subsequently proceeded to linear accelerator (LINAC) stereotactic radiosurgery (SRS) treatment of the bAVM, with the goal of complete occlusion. At six months post-SRS, the clinical improvements experienced after the treatment of hydrocephalus by VP shunting were maintained; however, there were not yet any radiological signs of bAVM occlusion. Further bAVM imaging will continue, as the latent interval between treatment and occlusion may be as long as three to five years.

## Discussion

Cerebral fluid dynamics

The Monro-Kellie doctrine assumes that the volume of the contents of the cranial vault (brain, blood, and CSF) remains constant [11]. Therefore, any alteration in any one of these components will require a compensatory increase or decrease in the other components to maintain a steady state. As a result, with every cardiac contraction, the volume of arterial inflow during systole causes an increase in intracranial blood volume requiring compensatory venous outflow and CSF displacement to the spinal subarachnoid space [12].

The presence of a large venous varix obstructing the cerebral aqueduct in this patient resulted in obstructive hydrocephalus, which impaired intracranial hemodynamics through obstruction to the bulk flow of CSF [7].

Hemodynamic consequences of obstructive hydrocephalus due to an unruptured brain arteriovenous malformation

Instead of low-resistance arteriovenous shunting and a reduction in PI that becomes more pronounced with larger bAVMs [13], this patient had a paradoxical hemodynamic effect whereby (1) PI was increased, (2) cerebral arterial flow within the MCAs was decreased, and (3) marked venous alterations were demonstrated following the insertion of the shunt.

The use of the pulsatility index using transcranial Doppler positively correlates to intracranial pressure, that is, an increase in the pulsatility index occurs with increasing intracranial pressure [14-15] caused by a reduction of brain tissue compliance compressing the arteriolar resistance vessels, resulting in an increase in arterial resistance. Although the reliability of the pulsatility index has been challenged as a primary diagnostic tool by some researchers [16-17], it remains a readily repeatable tool with a probability-based interpretation [16] and provided a useful parameter in demonstrating hemodynamic alterations in this case study. Normally, patients with bAVM have PIs ranging from as low as 0.14 to 0.88 in feeding arteries and 0.53 to 1.11 in ipsilateral non-feeding arteries [13]. In this case study, measurements were only taken from the MCAs, as this patient had a poor bony window. This also allowed the consistent identification of vascular structures to facilitate the demonstration of postoperative hemodynamic changes. Although the MCAs were not the feeding arteries in this case, one might predict (in the absence of this patient having obstructive hydrocephalus) a slight reduction in the PIs given their relative position in relation to the bilateral posterior cerebral artery feeding arteries. The left MCA’s markedly higher PI of 1.4 was likely due to a more localized compression effect from the obstruction of the bulk flow of the CSF within the subarachnoid space.

The velocities within the MCAs were also manifestly low as a result of decreased intracranial compliance giving rise to an increase in vascular impedance [18]. A minor improvement in the MCA velocities was evident two hours following shunt insertion and there was further improvement of the right MCA velocities at three months although the left MCA velocities were further reduced through unknown mechanisms.

The most likely explanation for the TCCD appearances of retrograde flow prominence and increased pulsatility after surgery is the relief of elevated CSF pressures. These findings are consistent with those of Bateman, which demonstrated a 186% increase in venous pulsatility in seven patients with normal pressure hydrocephalus (NPH) following shunt insertion [19]. In NPH, the proposed underlying causative mechanism is that of elevated cortical venous pressure [19] and decreased intracranial compliance [7]. In NPH, the mean CSF pressure is not elevated despite the ventricles being dilated [19]. However, in obstructive hydrocephalus, the elevated CSF pressure and ensuing ventricular dilatation cause acute venous stasis [7], hence, the condition has been referred to as “venous congestion hydrocephalus” [20]. The TCCD appearances, in this case, demonstrate that following shunt insertion, the veins are able to readily dilate and restore venous outflow.

Using transcranial color duplex, this case study illustrates the intracranial compliance changes that are associated with obstructive hydrocephalus and supports Greitz’s theories that following shunt insertion, intracranial vascular compliance is increased, resulting in the dilatation of the previously compressed capacitance vessels and restoration of venous caliber [7].

## Conclusions

We report the first TCCD assessment of the hemodynamic changes in the intracranial circulation in a patient with bAVM following ventricular-peritoneal shunting. Following shunt insertion, TCCD successfully demonstrated an increase in intracranial compliance, resulting in dilatation and an increase in the pulsatility of the previously compressed venous capacitance vessels. The results lend conceptual support to the theory that a high-pressure gradient change occurs in the veins as compared to the subarachnoid space. Furthermore, color duplex ultrasound was successfully used to demonstrate shunt patency in the neck.
