# Correction: Obstructive Hydrocephalus Due to Unruptured Brain Arteriovenous Malformation: Demonstrating Transcranial Color Duplex Confirmation of Cerebral Venous Hemodynamic Alterations and Color Duplex Ultrasound Confirmation of Shunt Patency

**DOI:** 10.7759/cureus.c26

**Published:** 2020-01-06

**Authors:** Kathryn J Busch, Hosen Kiat, Alberto Avolio, Mark Butlin, Andrew Davidson

**Affiliations:** 1 Miscellaneous, Faculty of Medicine and Health Sciences, Macquarie University, Sydney, AUS; 2 Cardiology, Faculty of Medicine and Health Sciences, Macquarie University, Sydney, AUS; 3 Faculty of Medicine, University of New South Wales, Sydney, AUS; 4 Cardiovascular Research, Department of Biomedical Sciences, Faculty of Medicine and Health Sciences, Macquarie University, Sydney, AUS; 5 Cardiology, Department of Biomedical Sciences, Faculty of Medicine and Health Sciences, Macquarie University, Sydney, AUS; 6 Neurosurgery, Faculty of Medicine and Health Sciences, Macquarie University, Sydney, AUS

The following errors have been corrected (all located in the three paragraphs between Figures 6 and 7 in the Case Presentation section):

First paragraph: "(Figure 4C)" reference removed and "(Figure 6A, 6B)" reference added.

Second paragraph: "Pressure support ventilation" replaced with "peak systemic velocity", "volume" replaced with "velocity", and "(Figure 7)" reference added.

Third paragraph: "VM" replaced with "mean velocity" and "(Figure 7)" reference removed.

